# Thermo-Mechanical and Morphological Properties of Polymer Composites Reinforced by Natural Fibers Derived from Wet Blue Leather Wastes: A Comparative Study

**DOI:** 10.3390/polym13111837

**Published:** 2021-06-01

**Authors:** Alessandro Nanni, Mariafederica Parisi, Martino Colonna, Massimo Messori

**Affiliations:** 1 Department of Civil, Chemical, Environmental and Material Engineering, University of Bologna, Via Umberto Terracini 28, 40131 Bologna, Italy; mariafederica.paris2@unibo.it (M.P.); martino.colonna@unibo.it (M.C.); 2 INSTM, National Consortium of Material Science and Technology, Via Giuseppe Giusti 9, 50121 Firenze, Italy; massimo.messori@polito.it; 3 Dipartimento di Scienza Applicata e Tecnologia, Politecnico di Torino, Corso Duca degli Abruzzi 24, 10129 Torino, Italy

**Keywords:** natural fibers, reinforcing fibers, polymer composites, electron microscopy, creep modelling, poly(lactic acid), polyammide12, thermoplastic elastomer, thermoplastic polyurethane

## Abstract

The present work investigated the possibility to use wet blue (WB) leather wastes as natural reinforcing fibers within different polymer matrices. After their preparation and characterization, WB fibers were melt-mixed at 10 wt.% with poly(lactic acid) (PLA), polyamide 12 (PA12), thermoplastic elastomer (TPE), and thermoplastic polyurethane (TPU), and the obtained samples were subjected to rheological, thermal, thermo-mechanical, and viscoelastic analyses. In parallel, morphological properties such as fiber distribution and dispersion, fiber–matrix adhesion, and fiber exfoliation phenomena were analyzed through a scanning electron microscope (SEM) and energy-dispersive spectroscopy (EDS) to evaluate the relationship between the compounding process, mechanical responses, and morphological parameters. The PLA-based composite exhibited the best results since the Young modulus (+18%), tensile strength (+1.5%), impact (+10%), and creep (+5%) resistance were simultaneously enhanced by the addition of WB fibers, which were well dispersed and distributed in and significantly branched and interlocked with the polymer matrix. PA12- and TPU-based formulations showed a positive behavior (around +47% of the Young modulus and +40% of creep resistance) even if the not-optimal fiber–matrix adhesion and/or the poor de-fibration of WB slightly lowered the tensile strength and elongation at break. Finally, the TPE-based sample exhibited the worst performance because of the poor affinity between hydrophilic WB fibers and the hydrophobic polymer matrix.

## 1. Introduction

Leather processing is one of the oldest and most widespread industries in the world. Around 1.7 billion m^2^ of leather are currently produced globally, with an estimated market price of around 34 billion euros. The main leather production centers are located in Asia, especially in China, followed by India and Japan, while among the European countries, Italy is the leader in this sector [1].

Finished leather is obtained by the treatment of raw hides and skins with different substances and reagents (such as tanning agents) through a process that mainly involves three steps, pre-tanning, tanning, and post-tanning, followed by various finishing operations before the materials are placed on the market. Leather processing involves the use of several chemicals, in addition to the chromium salts used for tanning, such as lime, sodium sulfite, ammonium salts, and various mineral acids, thus creating an enormous amount of liquid and solid wastes in the environment [1]. It is estimated that the processing of 1 ton of raw leather generates about 600 kg of solid waste and 50 m^3^ of wastewater [2].

About 30% of the entire amount of solid waste within the tanning process consists mostly of wet blue (WB) leather generated from the first tanning process [3]. The main problem regarding such waste is definitely the presence of chromium coming from the chromium salts used as tanning agents. For several years, landfilling has been the most used disposal method chosen by tanneries. However, this option is no longer practicable because of its environmental impact and the increasing contamination of soil and groundwater. In particular, what is of greatest concern is the possible oxidation, catalyzed by various factors such as humidity and temperature, of trivalent chromium contained in salts to hexavalent chromium, which is known to be hazardous and toxic for living beings and the environment [4].

Therefore, to avoid any possible consequences due to the degradative effects of these materials arising from their landfilling, researchers from all over the world are studying safer waste disposal methods in order to recover and reuse these wastes in other various industrial applications, such as footwear, fashion accessories, the automotive industry, and buildings. Because of the concerns about the environment and the sustainability of the plastic industry, these sectors are increasingly needing more green materials, and the use of natural reinforcing fibers would represent a way to reduce the carbon footprint in a cost-advantageous and feasible way, as testified by the growing interest in polymer bio-composites in both academic and industrial fields [5]. Beyond well-known lignocellulosic fibers such as hemp, kenaf, bamboo, and jute [6], researchers are also trying to valorize and transform other unconventional, non-food, competitive, and renewable agro-industrial crops into new bio-fillers or bio-fibers for the plastic sector. Examples include spent coffee grounds [7], wine [8,9], eggshell and seashell [10], tea [11], rapeseed [12], and coconut [13] wastes or by-products.

Therefore, from this perspective and because of their intrinsic fibrous nature and renewability, leather scraps could be successfully used as reinforcing fibers in polymer composite materials. Nevertheless, most of the works present in the literature have mainly focused on the use of hides and skins from post-consumer products or from mixed-leather wastes (wet blue, finished hides, or buffing/shaving dusts) with polymeric matrices from fossil sources [1]; meanwhile, there are few publications based on the exclusive use of wet blue wastes in composite materials [14,15,16,17]. Moreover, only two of these cited papers have used thermoplastic matrices (polyamide/polyethylene terephthalate [15] and poly(vinyl butyral [16])), and the reported characterization is not detailed enough to provide and assess the behavior of WB leather used as reinforcing fibers.

Therefore, this work aimed to test WB scraps as reinforcing fibers within several polymer matrices such as polyamide 12 (PA12), poly(lactic acid) (PLA), thermoplastic polyurethane (TPU), and a thermoplastic elastomer (TPE) to offer a wide screening of the effect of these natural fibers on different matrices in terms of thermal, rheological, thermo-mechanical, viscoelastic, and morphological properties.

From an applicative and industrial point of view, PLA is selected in order to decrease its production costs without altering its bio-based content. In fact, PLA is a fully bio-based and biodegradable polymer that, despite its outstanding properties, is scarcely present in the market, because of its high price [18,19]. Similarly, PA12, TPU, and TPE are selected because they are currently widely used in the wearables sector, in particular footwear applications [20,21,22], such as shoe inserts and soles, in which finished leathers can be used, too. The possibility of using plastic materials containing WB wastes in combination with finished leather is surely an attractive option since it meets the main principles of circularity within the same productive chain [23].

## 2. Materials and Methods

### 2.1. Materials

Polyamide 12 (PA12; Grilamid^®^ XE3982 grade) and Marfran (TPE) KP330 (a thermoplastic elastomeric blend of styrene-ethylene-butylene-styrene and polyolefin) were provided by EMS-Grivory (Domat/Ems, Switzerland) and Marfran s.r.l. (Nigoline (BS), Italy), respectively. Poly(lactic acid) (PLA; Ingeo^TM^ 4043D grade) and thermoplastic polyurethane (TPU; Elastollane 11600 D50 grade) were purchased from NatureWorks LLC (Minnetonka, MN, USA) and BASF (Ludwigshafen, Germany), respectively.

**Figure 1:**
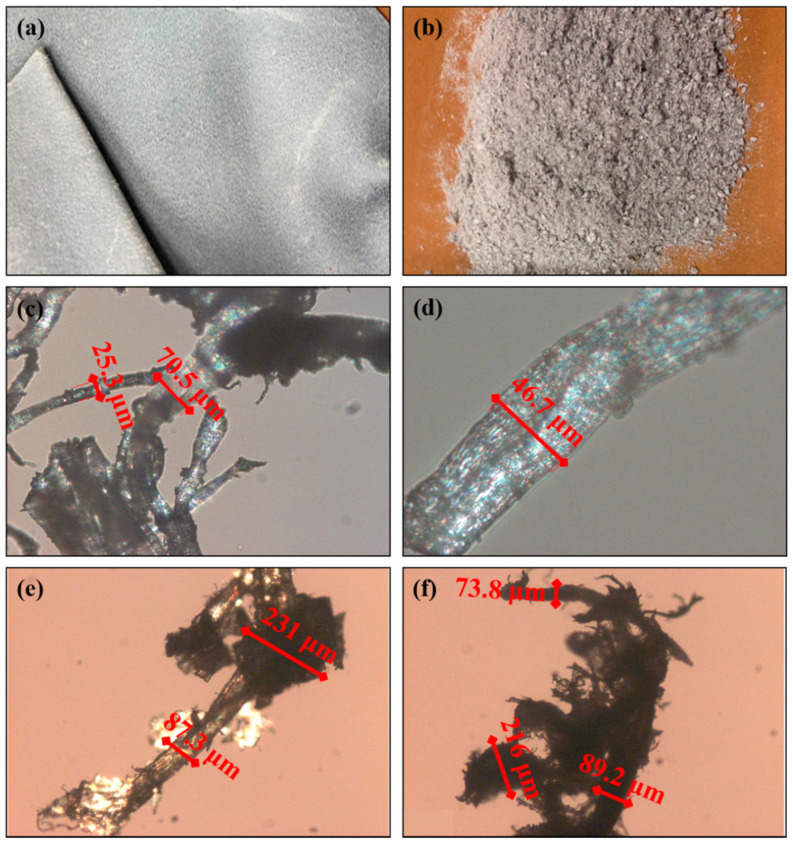
Pictures of the WB leather before (**a**) and after (**b**) grinding and images of the grinded WB fibers taken by an optic microscope (**c**–**f**). It is possible to recognize three different structures: the main fibrous body (mean diameter of 70 μm), branched and exfoliated tiny fibers (22 μm), and bulky and coarse entangled fiber clusters (222 μm).

Wet blue (WB) scraps (Figure 1a) were provided by Sciarada s.p.a. (Pisa, Italy). These were ground into fibers (Figure 1b) by the means of an industrial blade granulator (C.M.G., model N17-22-3K).

### 2.2. Characterization of the WB Fibers

The apparent density ($\rho_{app}$) of the ground WB fibers was measured by reading the volume occupied by 2.1 g of WB fibers in a 50 mL graduated cylinder. The real particle density ($\rho_{real}$) was calculated the same way, with the only difference that WB fibers were pressed by a 73 g weight made of steel. The moisture content of WB fibers was determined by oven-drying 500 g of a sample until it reached a constant weight. The morphological shape of the WB fibers was evaluated by taking images with an optical microscope (SOM Wild M3 Heerbrugg), while the chemical composition was evaluated through infrared spectroscopy in ATR configuration using a Perkin-Elmer UATR Two spectrometer. The reported spectrum was the average of 16 scans with 4 cm^−1^ resolution in the 4000–400 cm^−1^ range. Finally, the thermal stability of dried WB fibers was investigated through thermogravimetric analysis (TGA; Perkin-Elmer TGA4000) equipped with an infrared detector (Perkin-Elmer Spectrum One) in order to simultaneously evaluate the structure of the degradation products. TGA tests were also conducted under isothermal conditions (200 °C) for 10 min to evaluate the maximum processing residence time within the internal mixer.

### 2.3. Preparation of the Composites

Polymer pellets were melt-mixed with 10 wt.% of WB fibers using an internal mixer (Brabender) operating at 50 rpm. The mixing process was conducted for 6 min, and the temperature was set to 180 °C for PA12- and PLA-based composites and 190 °C for TPE- and TPU-based formulations. During this phase, the torque was recorded as a function of time to evaluate the processability of the investigated systems. Compounded materials were then ground into new pellets that were subsequently used as feed for a MegaTech Tecnica DueBi injection-molding machine to obtain specimens for tensile property evaluation (type 1BA, ISO 527). Before each processing step, both polymer pellets and grounded WB fibers were oven-dried at 80 °C for 4 h. Neat polymers processed under the same conditions of composites were labeled with their common acronyms (PLA, PA12, TPU, and TPE), while composites materials were labeled as PLA10WB, PA1210WB, TPU10WB, and TPE10WB.

### 2.4. Characterization of the WB-Based Composites

#### 2.4.1. Thermal Properties

Thermal properties of WB-based composites were evaluated by differential scanning calorimetry (DSC) using a Perking-Elmer DSC 6 apparatus under nitrogen flow (20 mL min^−1^). Each sample (10 ± 3 mg) was first heated from room temperature to 220 °C at a heating rate of 20 °C min^−1^ to erase the previous thermal history and was subsequently cooled to −40 °C at a heating rate of 20 °C min^−1^ to determine the crystallization temperature (T_C_) and the crystallization enthalpy (*H_C_*). Finally, the samples were heated again to 220 °C at a heating rate of 20 °C min^−1^ and the melting temperature (T_m_) and melting enthalpy (*H_m_*) were evaluated. When present, the glass transition temperature (T_g_), post-crystallization temperature (T_pc_), and post-crystallization enthalpy (*H_pc_*) were also determined from the second heating cycle. For the PLA- and PA12-based formulations, the crystallinity percentage (χ) was calculated as

(1) $$\chi =\left(\frac{H_{m}-H_{pc}}{H_{Ref}\times w_{p}}\right)\times 100,$$

where $H_{m}$ and $H_{pc}$ are the melting and post-crystallization enthalpies, respectively, of the samples evaluated during the second heating cycle; $w_{p}$ is the polymer weight fraction; and $H_{ref}$ is the melting enthalpy of the fully crystalline polymer. The $H_{ref}$ of PLA and PA12 were considered to be 93 J g^−1^ [24,25] and 209.2 J g^−1^ [26,27], respectively. In the case of TPE- and TPU-based composites, because of the lack of information concerning the $H_{ref}$ of the neat polymers, the variation in crystallinity due to the addition of WB fibers was calculated as follows:

(2) $$\Delta \chi =\left(\frac{\chi_{c}}{\chi_{p}}-1\right)\times 100=\left(\frac{H_{m,c}}{H_{m,p}\times w_{p}}-1\right)\times 100,$$

where $H_{m,c}$ and $H_{m,p}$ are the melting enthalpies of the WB-based composite and the neat polymer, respectively, evaluated during the second heating cycle.

#### 2.4.2. Thermal Stability

Thermal stability of the WB-based composites (and of neat WB) was investigated trough TGA using a Perking-Elmer TGA4000 apparatus purged by a 40 mL min^−1^ nitrogen flow. TGA tests were conducted on 10 ± 2 mg of samples using a ramp temperature from 50 °C to 600 °C, increasing at a heating rate of 10 °C min^−1^. From each thermogram, the temperatures at 5 and 15 wt.% loss (T_5_ and T_15_) and degradative peak temperatures (T_peak_) were determined. T_peak_ is the temperature at which the maximum derivate of the weight with respect to temperature (DTA) is observed.

#### 2.4.3. Contact Angle

The water contact angle was evaluated using a drop shape analyzer (DSA30S- Kruss), and each reported value was the average of at least five measurements taken on different locations of the samples.

#### 2.4.4. Mass Melt Flow Rate

The melt mass flow rate (MFR) was determined according to ISO 1133 (part II) by means of MFI AMSE XNR 400 C. Tests were conducted using a load of 2.16 kg and a temperature of 210 °C.

#### 2.4.5. Mechanical Properties

Tensile tests were performed using an INSTRON 5966 dynamometer equipped with a 10 kN load cell. Tests were conducted at room temperature using a clamp separation speed of 50 mm min^−1^ for each formulation, except PLA-based composites, which were tested with a speed of 5 mm min^−1^. The Young modulus (E), tensile strength (TS), and elongation at break (ε_b_) were reported as an average of at least five determinations. Finally, the impact properties were evaluated by means of an AMSE Model XJJD Charpy Impact tester in accordance with the ISO 179 standard.

#### 2.4.6. Viscoelastic Properties

The viscoelastic behavior of the WB-based composites was measured using a TA DMA Q800 instrument operating in single-cantilever configuration. Tests were run from −30 °C to 90 °C at a heating rate of 4 °C min^−1^, an oscillating frequency of 1 Hz, and an applied strain of 0.1%. The storage modulus (E’) and damping factor (tan δ) were plotted as a function of temperature, while the glass transition temperature (Tg) was determined as the temperature at which the maximum value of tan δ was exhibited.

Creep and recovery strain tests were conducted at 25 °C using a TA DMA Q800 instrument operating in single-cantilever configuration. Creep compliance was recorded by loading the specimens with a 1 MPa stress for 10 min, while the recovery step was of 15 min duration.

To evaluate in detail the elastic and time-dependent viscous contributions of both creep and recovery phases, 4-parameter Weibull-like functions [28] were fitted on experimental data using the software OriginPro 7.5. When applied to the creep data, the mentioned function, also known as the Kohlrausch–Williams–Watts (KWW) model [29], has the following form:

(3) $$J\left(t\right)=J_{E}+J_{V}\left\{1-exp\left[-{\left(\frac{t}{t_{c}}\right)}^{\beta_{c}}\right]\right\},$$

where $J_{E}$ and $J_{V}$ are the instantaneous elastic and the viscous creep compliance contributions, respectively, while $t_{c}$ and $\beta_{c}$ are the characteristic time and the shape parameter of the creep compliance, respectively.

When applied to creep recovery data, the equation maintains its form but the terms are different, and it has the following form:

(4) $$R\left(t\right)=R_{E}+R_{V}\left\{1-exp\left[-{\left(\frac{t}{t_{c}}\right)}^{\beta_{c}}\right]\right\},$$

where $R_{E}$ and $R_{V}$ are the instantaneous elastic and the viscous creep recovery, respectively, and they are expressed as a percentage of the recovered strain with respect to the maximum deformation observed at the end of the creep test. Again, $t_{c}$ and $\beta_{c}$ represent the characteristic time and the shape parameter of the creep recovery, respectively.

These models, derived from the consideration that viscoelastic changes in polymers are due to molecular incremental jumps between different position of relative equilibrium [28,29], have been successfully applied to creep data and recovery data of several different composites [30,31]. Moreover, these models can also be exploited to predict the creep compliance and creep recovery for times longer than the ones used for the tests. Indeed, the sum of $J_{E}$ and $J_{V}$ as well as that of $R_{E}$ and $R_{V}$ represent the viscous creep compliance ($J_{\infty }$) and the creep recovery percentage ($R_{\infty }$), respectively, for times tending to infinite, while $t_{c}$ represents the time at which 63% of the viscous creep compliance, or analogously the creep recovery, is reached.

#### 2.4.7. Morphology

The morphology, adhesion, distribution, and dispersion of WB fibers within the polymer matrices were observed with a scanning electron microscope (SEM-FEG MIRA3-TESCAN) equipped with a microanalysis X-EDS Bruker XFlash 630M system. Briefly, rectangular specimens were broken in liquid nitrogen, and the cross section was covered by a 10-nm-thick layer of graphite. The obtained surfaces were observed with the SEM operating under high-vacuum conditions and with an electron beam of 20 kV.

## 3. Results and Discussion

### 3.1. Characterization of the WB Fibers

The main properties of the wet blue (WB) fibers used in this work are reported in Table 1. From a morphological point of view, WB fibers presented a fibrous backbone with a mean size diameter of around 70 μm, which branched into tiny exfoliated fibers (22 μm) and entangled quasi-spherical coarse clusters (222 μm) along the edge of the fiber backbone (Figure 1). Despite the simultaneous presence of these three different structures, the WB fibers had overall good homogeneity in terms of shape and mean diameters. The presence of branched and rough tiny fibers is a positive feature since they have higher chances of mechanically interlocking with the polymer chains during the melt compounding phase. However, the presence of coarse clusters could negatively affect the mechanical performances of composites since they could inhibit or limit the correct orientation of the fibers during the injection molding process, leading to a not-oriented, less resistant composite product [32,33]. Finally, the density of the WB fibers (Table 1) was low (apparent and real densities of 190 and 300 g dm^3^, respectively), evidencing the possibility to obtain lightened composite materials.

**Figure 2:**
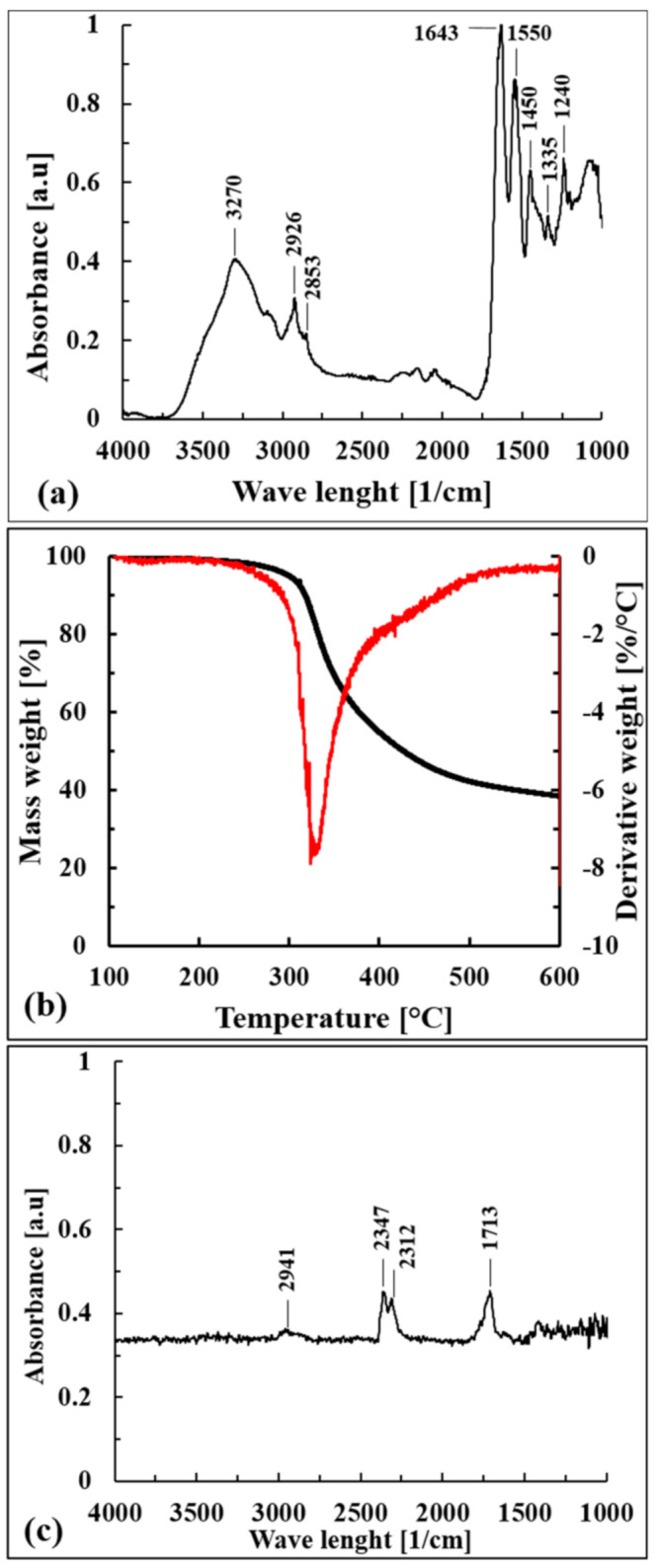
(**a**) ATR spectrum of WB fibers, (**b**) TGA and DTA curves of dried WB fibers, and (**c**) FT-IR spectrum of degradative products released by WB fibers at T_peak_ (328.6 °C).

From a chemical point of view, the FT-IR (ATR) spectrum shown in Figure 2a confirmed that the composition was due to bovine skins, which consist mostly of water and proteins such as collagen (29%), keratin (2%), and elastin (0.3%) and minor components such as fats and inorganic substances [34]. It is possible to recognize the typical signals of NH and OH stretching vibrations (3300–3270 cm^−1^), while the peak at 2926 cm^−1^ is ascribed to the stretching of the CH group. The amide I vibration, adsorbing near 1650 cm^−1^, is ascribed to C=O stretching along with C-N stretching and N-H bending.

The signals near 1550 cm^−1^ are ascribable to the presence of secondary amides; in particular, they arise from NH bending and CN stretching, with a smaller contribution of CO bending. The peaks in the region from 1400 to 1200 cm^−1^ are ascribed to the presence of tertiary amides; they are associated with NH bending and CN stretching, with a smaller contribution of CO bending and CC stretching.

Dried WB fibers showed an intrinsic pronounced thermal stability, as confirmed by the thermogravimetric data reported in Table 1 and by the curves shown in Figure 2b. WB fibers degraded in one mass loss step between 300 °C and 330 °C, and the FT-IR spectrum of released gases at T_peak_ (328.6 °C) is reported in Figure 2c.

The asymmetrical stretching bands of CO_2_ (2312–2347 cm^−1^), the C=O carbonyl stretching band (1713 cm^−1^), and the CH stretching band (2941 cm^−1^) can be detected. These reveled peaks could be associated to the direct decarboxylation of free –COOH groups of carboxylic acids, generally present in high amounts in bovine collagen [35], that would have formed carbon dioxide and/or to the reactions of dehydration of intramolecular amino acids [36] that would have mainly formed degradative products with carbonyl groups. The CH stretching absorption could be attributed to the formation of hydrocarbons, probably ethane [36], and/or to the degradation of organic products containing carbonyl groups. Nevertheless, further works should confirm these hypotheses through mass spectrometry, which is more capable for the purpose.

From a technological point of view, WB fibers were also found stable in isothermal conditions (200 °C) for around 10 min, evidencing the possibility of processing these natural fibers within internal-mixer or twin-screw extruders for significant residence times without encountering degradation.

### 3.2. Processability of the WB-Based Composites

**Figure 3:**
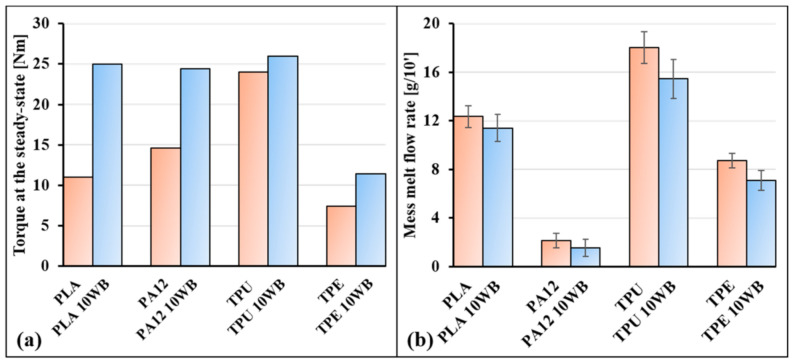
(**a**) Torque values recorded at the steady state during the internal-mixer processing and (**b**) mass melt flow rate (MFR) values of the WB-based samples.

The processing behavior of WB-based composites during the melt-mixing step was investigated, recording the torque as a function of time. Generally, the torque rapidly increases when the polymer and fibers are added to the internal mixer, since polymer pellets are still in the solid state, and subsequently, the torque starts to decrease as a consequence of the melting or softening of the polymer matrix. After a certain time (generally 4–10 min), which depends on the processing conditions such as temperature and screw speed and on the polymer and fiber typologies, the torque reaches a steady state and a stable value, indicating that the entire polymer has melted (or softened) and fibers (or fillers) have been well dispersed and distributed in the polymer matrix [37]. The equilibrium values of the torque (T) of the WB-based samples are reported in Figure 3a, where it can be noted that the addition of 10 wt.% of WB fibers increased the T values of respective neat polymers. This expected phenomenon is explainable by the fact that WB fibers increase the melt viscosity of the polymer and thus the torque value, as similarly reported in other works [38,39,40]. Nevertheless, from a comparative point of view, the increase in torque for PA12- and, especially, PLA-based composites is much higher than for TPE- and TPU-based formulations. Since PLA and PA12 present polar groups in the polymer backbone, their interactions with polar WB fibers are much stronger than in the case of TPE- and TPU-based samples, and the required mixing energy is therefore significantly higher. It is reasonable to believe that this pronounced affinity exhibited during the compounding step would be positively reflected in the morphological and mechanical properties of the PLA10WB sample.

The increased melt viscosity of WB-based composites was also testified by the mass melt flow rate (MFR) values reported in Figure 3b, from which it can be observed that MFR values of polymers decreased by around 10–20% by WB addition, similarly to other works on composites [41]. In parallel, as reported in Table 2, the thermal stability of neat polymers was lowered by WB fibers by around 5–30 °C. This result was expected, since natural fibers or fillers generally lower polymer stability [42] and thus their resulting composites start to degrade at lower temperatures. Nevertheless, from a technological point of view, both increases in viscosity and decreases in thermal stability did not significantly affect the injection molding processing, since it was conducted under the same conditions of neat polymers, at temperatures well below the degradative temperatures (T_5_ and T_15_).

### 3.3. Thermal Properties of the WB-Based Composites

**Figure 4:**
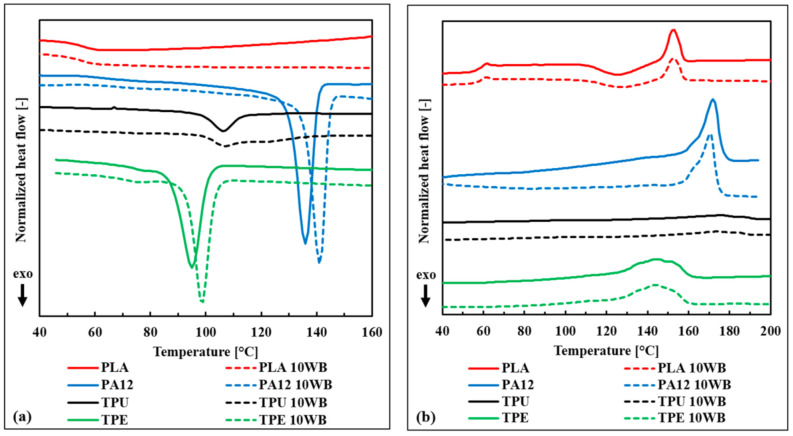
DSC curves of WB-based samples during (**a**) cooling and (**b**) the second heating scan.

The thermal properties of the WB-based composites, evaluated trough DSC, are reported in Table 3 and in Figure 4. From these data, it is evident that both crystallization (T_c_) and melting (T_m_) temperatures were not significantly influenced by the addition of WB fibers. The only detected slight differences between composites and corresponding neat polymers were regarding the crystallinity values (χ). In particular, the TPU10WB sample exhibited a lower crystallinity (−4.3%) compared with neat TPU. This fact indicates that WB fibers could have hindered the self-assembly of the hard blocks of TPU into crystalline domains during the cooling cycle. In contrast, PA12- and TPE-based composites showed enhanced crystallinities (+1.5% and +1.0%, respectively) and higher crystallization temperatures (+4.9 and +3.6 °C, respectively), indicating the ability of WB fibers to work as a nucleating agent under certain conditions, in agreement with what was observed in other works dealing with fiber-reinforced composites [25,43].

### 3.4. Mechanical and Morphological Properties of the WB-Based Composites

**Figure 5:**
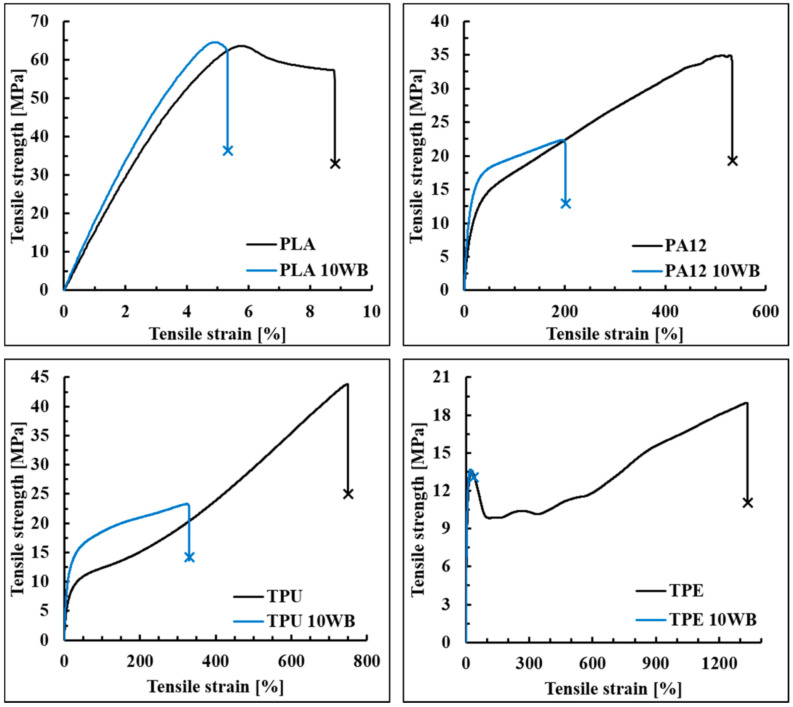
Tensile behavior of WB-based composites.

In Table 4, the tensile and impact properties of the WB-based composites are summarized. It can be noticed that WB fibers improved the Young modulus of the investigated polymers from around 20%, in the case of PLA and TPE, up to 36% and 58%, in the case of PA12 and TPU, respectively. This pronounced gain can be explained by the higher stiffness of the WB fibers if compared to the polymer matrices. In addition, since the elastic modulus of composites is dependent on the volume fraction of the added fiber/filler (and not on their weight fraction), the lightness of the WB fibers would explain the significant gain in stiffness despite the loading at just 10 wt.% (which in fact corresponds to 25–31% in volume). In contrast, tensile strength and elongation at break values were found lowered by the addition of WB fibers in all samples, except PLA10WB (Figure 5). In composites, these mechanical properties mainly depend on morphological aspects such as the distribution and dispersion of the fibers within the polymer matrix and the interfacial adhesion between the polymer and the fibers [32]. Generally, the distribution and dispersion of natural fibers is not easily estimated, both quantitatively and qualitatively, since they are mainly formed by cellulose, hemicellulose, and lignin, which have the same elemental composition as polymer matrices [44]. In this case, because of the presence of inorganic elements and compounds such as chromium, sodium chloride, and sulfur, it was possible to evaluate qualitatively the WB dispersion and distribution within the different polymer matrices through energy-dispersive X-ray spectroscopy (EDS).

**Figure 6:**
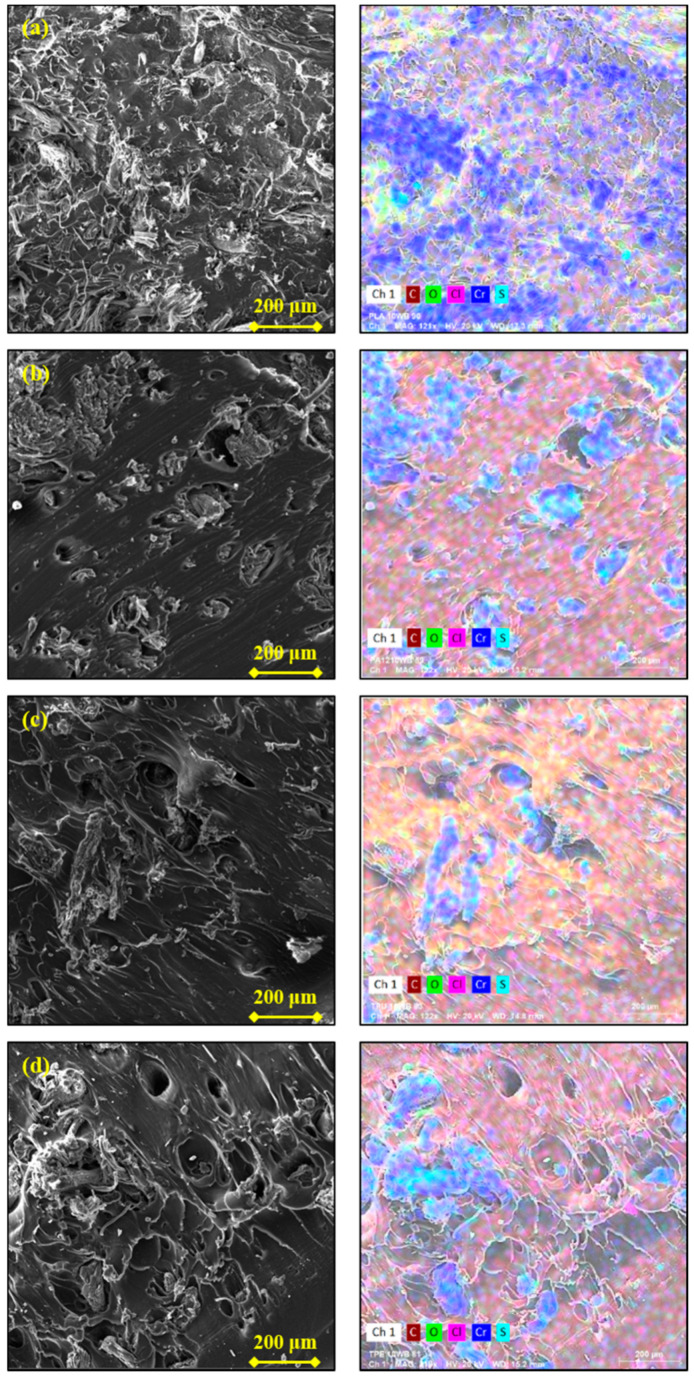
Scanning electron microscope (SEM) images of PLA- (**a**), PA12- (**b**), TPU- (**c**), and TPE-based (**d**) composites taken at low magnification (200×) and their corresponding EDS maps on the right.

The SEM images of the WB-based samples taken at low magnification (200×) and their corresponding EDS maps are reported in Figure 6. It is possible to notice that within TPU and TPE matrices, WB fibers formed aggregates and they were not homogenously distributed through the polymer matrix (Figure 6c,d). In contrast, the distribution and dispersion of WB fibers was still good within PA1210WB and excellent within PLA.

**Figure 7:**
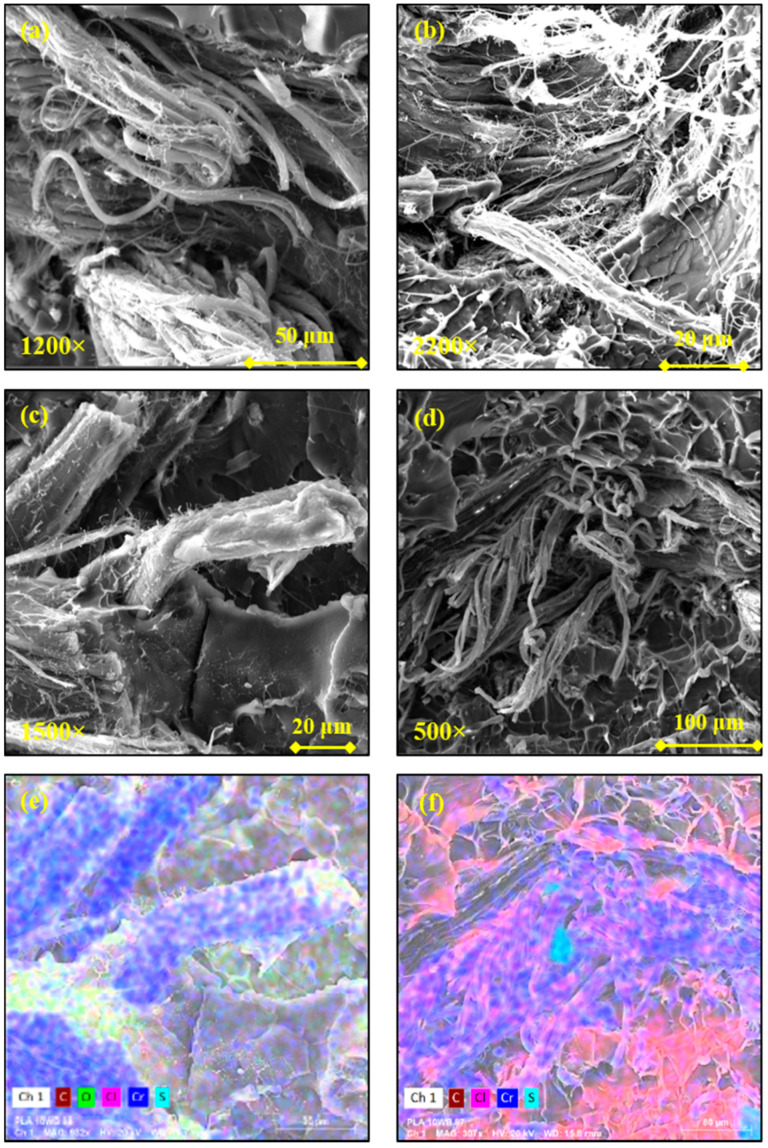
Scanning electron microscope (SEM) images of the PLA10WB sample at different magnifications (**a**–**d**); (**e**) EDS map of micrograph (**c**) and (**f**) EDS map of micrograph (**d**).

In the case of PLA, the excellent dispersion and distribution is also due to the fact that WB fibers appear de-fibered and branched in nanofibers, which therefore occupy the polymer matrix better and more uniformly. These tiny WB fibers are depicted in Figure 7, from which it can also be noticed how they are perfectly bonded with the polymer (Figure 7c), since the decrease in the fiber diameter enhances the fiber–matrix interface and the fiber wettability [45].

**Figure 8:**
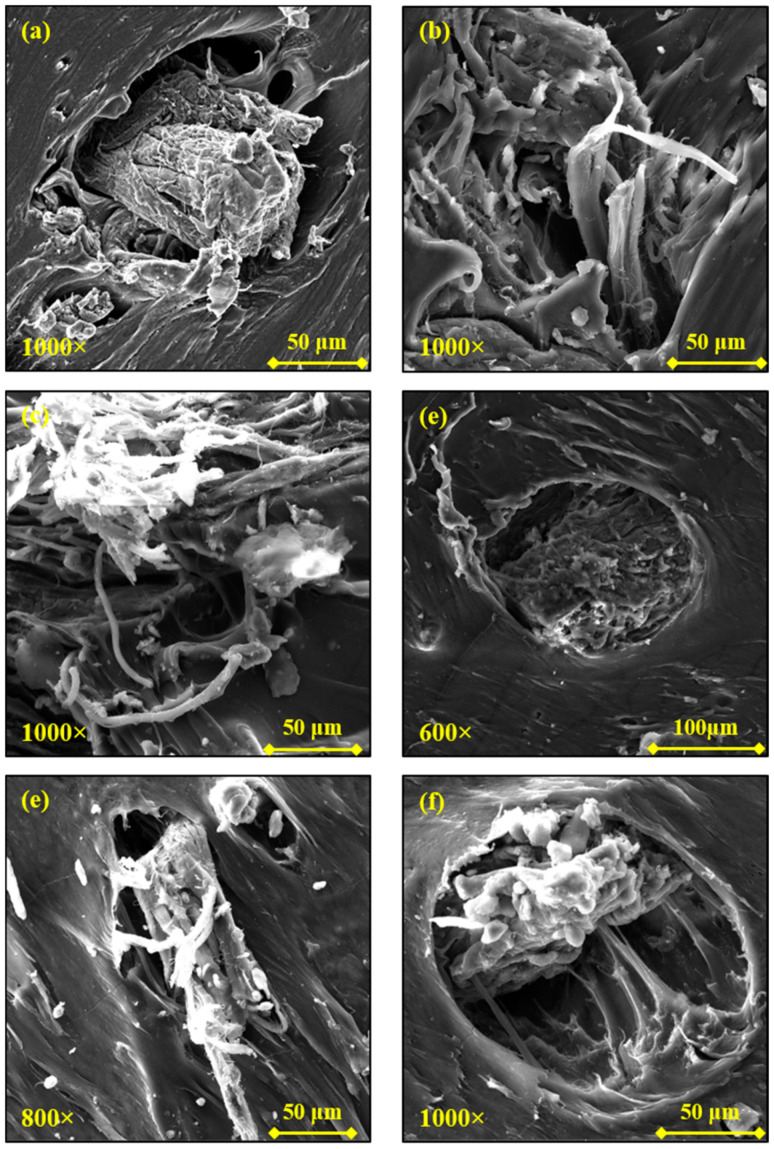
Scanning electron microscope (SEM) images of the PA1210WB (**a**,**b**), TPU10WB (**c**,**d**), and TPE10WB (**e**,**f**) samples at different magnifications.

This phenomenon, even if in a limited way, was also observable in PA12 composites (Figure 8a,b), while it was absent in the TPU- and TPE-based composites (Figure 8c–f). These observations are perfectly consistent with the torque analysis previously described: the higher torque values recorded during the processing of PA12- and, especially, PLA-based samples could have aided the de-fibering of the WB.

Nevertheless, cap-shaped cavities were also recognized within the PA1210WB surface, indicating that some WB fibers are poorly connected with the PA12 matrix. As a consequence of this poor polymer–fiber adhesion, the tensile strength (TS_max_) and elongation at break ($\varepsilon_{b}$) values of the PA1210WB sample were lowered by around 35% and 66%, respectively, compared with neat PA12. Analogously, TPU10WB showed a similar decrease in those properties (−44% and −57%) despite much fewer voids detected across its surface. As previously mentioned, TPU10WB did not present de-fibered WB. Therefore, it can be supposed that mechanical properties overly reflect and balance the concomitant presence of positive and negative morphological aspects, as reported in Table 5. Thus, the TPE10WB sample, which exhibited not-ideal WB dispersion and distribution and poor fiber–matrix adhesion (high presence of voids) and was not subjected to de-fibering effects, showed the worst mechanical performance, as confirmed by the dramatic loss in the elongation at break value (Table 4). In contrast, the PLA10WB composite showed enhanced tensile strength as a consequence of its excellent morphological properties.

From a chemical point of view, these morphological and mechanical behaviors are connected with the chemical structure of the investigated polymers. In particular, hydrophilic WB fibers are able to interact significantly with the polar ester groups of PLA to partially interact with the polar urethane groups of TPU [46] and with the polar amide linkages of PA12 [47] and to poorly join with non-polar TPE.

**Figure 9:**
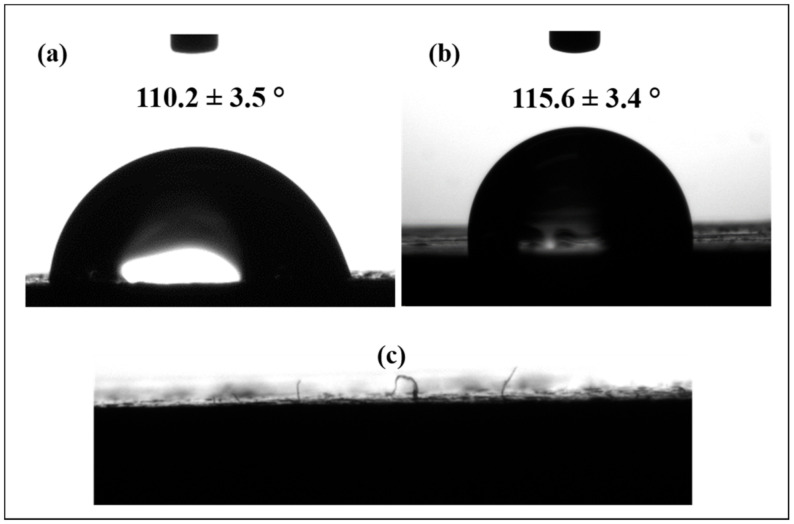
Water contact angle photos of (**a**) PLA10WB and (**b**) TPE10WB and (**c**) of the surface of the PLA10WB sample in which WB fibers are recognizable.

The water contact angle values of investigated samples are reported in Table 4. Considering and comparing neat polymers, the obtained values are in perfect agreement with the polarity of the polymer matrices, as previously discussed. Indeed, PLA showed the lower water contact angle (90.6°), followed by PA12 (98.9°), TPU (119.9°), and TPE (124.0°). In contrast, considering reinforced polymers, the addition of WB fibers increased the contact angle values of each polymer, except TPE. This fact was unexpected since hydrophilic fibers generally increase the surface wettability of polymers. Other authors [48,49] have reported a similar behavior investigating wood plastic composites formed by wood flours and/or fibers and polyolefins, but an explanation of the phenomenon was not furnished. Our interpretation is that the exfoliated nanofibers could have reached the polymer surface and, therefore, the contact angle values would have been increased because of this induced roughness on the polymer surface (Figure 9c). A similar explanation was also furnished in [50], where the insertion of glass fibers in a rubber matrix generated a morphological effect of roughening able to overcome the polar character of the SiO_2_ glass groups and thus to increase the surface hydrophobicity of the rubber. In addition, this hypothesis seems to be supported by the fact that the TPE10WB sample, which did not manifest de-fibering phenomena, showed a decreased water contact angle. Nevertheless, further works should investigate this possibility in depth.

Finally, the PLA10WB sample exhibited an enhanced impact resistance (+11%) compared with neat PLA, indicating the ability of WB fibers to also act as energy absorption fibers, similar to what has been observed in other PLA-based composites [51,52].

### 3.5. Viscoelastic Properties of the WB-Based Composites

**Figure 10:**
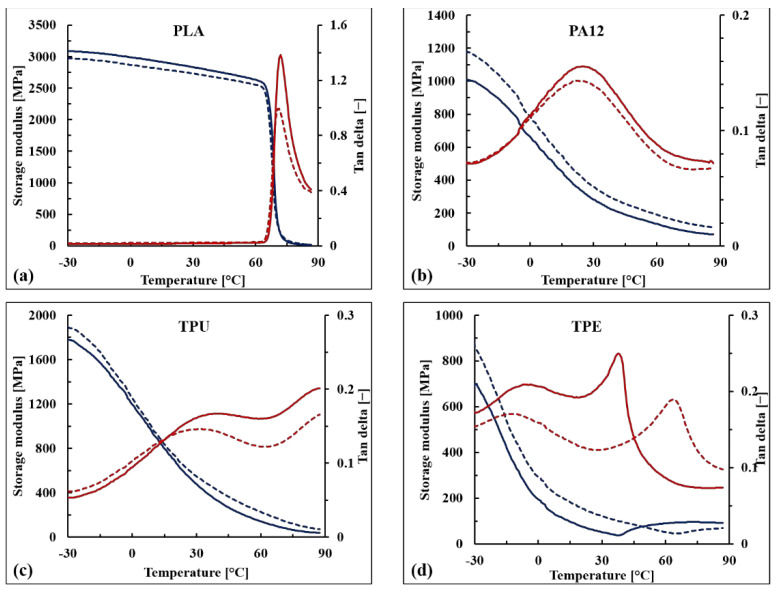
Dynamic mechanical analysis (DMA) of PLA (**a**), PA12 (**b**), TPU (**c**), and TPE (**d**) samples. Blue and red curves refer to the storage modulus (E’) and the damping factor (tan δ), respectively; meanwhile, the solid and dotted lines indicate the properties of the neat polymer and the corresponding WB-based composite, respectively.

The storage modulus (E’) and the damping factor (tan δ) of the WB-based samples are reported as a function of temperature in Figure 10 as well as in Table 6. Similar to what was observed for the tensile modulus, the stiffening effect of the WB fibers was confirmed for PA12-, TPU-, and TPE-based samples within the whole range of tested temperatures (from −30 to 90 °C), and the increments in E’ values were particularly marked above the glass transition temperature (T_g_) (+40–60%). This behavior was observed for each formulation, except PLA10WB, which exhibited slightly decreased E’ values compared with neat PLA. This contrasting behavior between storage and elastic moduli could be due to the orientation of the WB fibers that were oriented parallel to the applied force in the case of the tensile modulus, while in the case of DMA, the applied stress was perpendicular to the specimen axis and, thus, to the fiber orientation. This anisotropic behavior has also been observed in other composites reinforced by leather fibers [53].

Looking at the tan δ curves, it can be noticed that below the glass transition temperature, no significant difference between neat and reinforced polymers is observable. Above T_g_, WB-based composites exhibited lower tan δ values, except for TPE, indicating that reinforced samples have a more elastic than viscous nature compared with corresponding neat polymers [54]. The maximum damping reduction was observed in T_g_ as a consequence of the stiffness of the WB fibers that affect the relaxation of the neat polymer from the glassy to the rubbery state. Nevertheless, the T_g_ values of WB-based composites were not particularly affected, indicating that WB fibers did not immobilize the polymer chains [55].

The unique significant difference in terms of damping behavior was observed when comparing neat TPE with TPE10WB. From a qualitative point of view, the transitions of thermoplastic elastomers are not easy to discuss since they strongly depend on many factors such as the typology and amounts of the elastomeric component, of the polyolefin, and of the oils and additives involved in the formulation [56]. Regarding the TPE used in this study, the only information provided by the producer was that it is formed by SEBS and polyolefins. According to the literature [56], different peaks can be recognized in SEBS–polyolefin blends: the glass transition of the soft segment of a styrene–olefin block copolymer (from −70 to −55 °C), the glass transition of the amorphous domain of the polyolefin (from −20 to 10 °C), the α-relaxation of the crystalline phase of the polyolefin (between 50 and 125 °C), and the glass transition of the hard segment of the styrene–olefin block copolymer (between 30 °C and 60 °C).

Looking at the neat TPE curves (Figure 10d), only two different peaks of tan δ can be observed. The first peak, at around −7 °C, is attributed to the β-relaxation of the amorphous region of the polyolefin (polypropylene and/or polyethylene), while the second one, at around 40 °C, is attributed to the glass transition of the hard segment of styrene-olefin block copolymer. The first transition was not significantly affected by the addition of WB fibers, while the second one increased by around 20 °C. Therefore, it can be supposed that the WB fibers hinder and immobilize the hard segments of the TPE polymer, leading to a much more brittle material. This hypothesis is also supported by the dramatic loss in elongation at break values of TPE10WB, as previously shown.

**Figure 11:**
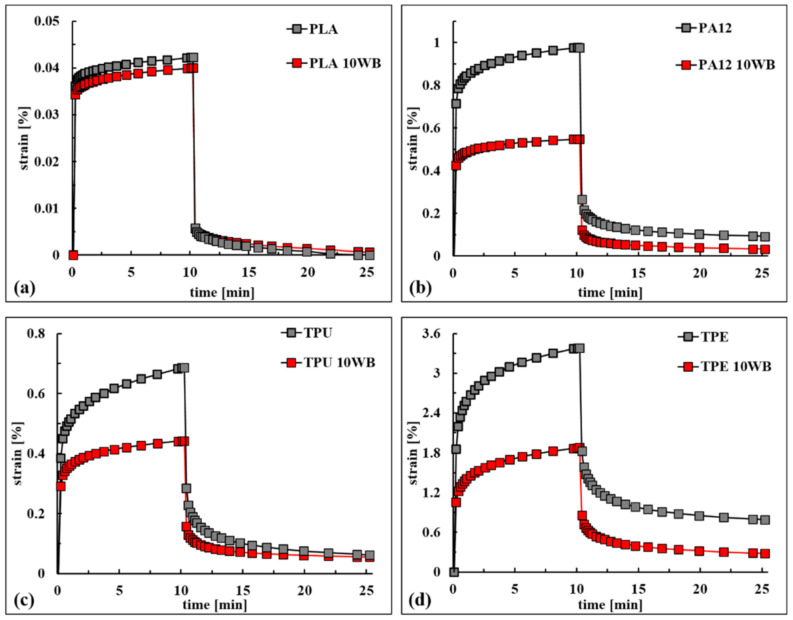
Creep deformation and strain recovery of (**a**) PLA-, (**b**) PA12-, (**c**) TPU-, and (**d**) TPE-based composites.

The creep deformations and strain recovery of the WB-based samples are reported in Figure 11. Generally, when a polymer is loaded below its elastic limit, there is an instantaneous elastic deformation, followed by a slower, time-dependent, viscoelastic deformation. When the load is removed, an instantaneous elastic recovery takes place, which is followed by a further time-dependent viscoelastic recovery, which may or may not be complete within the given limited time of the test. In this case, a permanent deformation is observable.

The maximum creep compliance ($J_{10^{\prime }}$) and the creep recovery ($R_{15^{\prime }}$) observed during tests, as well as the extrapolated parameter of the models, are reported in Table 7.

From an overall point of view, the $J_{10^{\prime }}$ values of PLA-, PA12-, TPU-, and TPE-based composites reduced0 by the addition of around 5%, 44%, 35%, and 44% WB fibers, respectively, compared with neat polymers. In addition, the lowered creep compliance was also maintained for time tending to infinity ($J_{\infty }$), indicating the ability of WB fibers to improve the creep resistance for both short and long times.

In the case of PLA, PA12, and TPE, WB fibers improved both elastic and viscous creep resistance in a comparable way since both $J_{E}$ and $J_{V}$ lowered by around 6%, 42%, and 44%, respectively, while in the case of the TPU10WB sample, WB fibers mainly lowered the viscous deformation (+62%) rather than the elastic one (+24%).

Comparing the elastic contribution to the maximum experimental creep compliance ($J_{E}$/$J_{10^{\prime }}$), it can be noticed that PLA- and PA12-based samples mainly deformed elastically (86% and 76%), while in TPE-based samples both contributions were more involved (56%). Considering the viscous creep compliance limit for times tending to infinity ($J_{\infty }$), it can be observed that it only slightly diverges from $J_{10^{\prime }}$ in the case of TPE and TPE10WB samples, as also confirmed by the characteristic time $t_{c}$, which was just 156 and 206 s. PA12-based samples and neat TPU deformed slower, as confirmed by the $J_{10^{\prime }}$/$J_{\infty }$ ratio, which was around 83%; meanwhile, TPU10WB reached 97% of the theoretical maximum creep compliance after 10′, since its creep response was mainly elastic. Finally, PLA-based samples showed the highest $t_{c}$ values, indicating that viscous deformations become significant only after long times of applied stress. In conclusion, WB fibers were able to improve the creep response of the investigated materials, reducing both elastic and viscous deformations. In the case of PLA- and TPU-based samples, the characteristics times were lowered by the addition of WB fibers, evidencing that these composites deform more rapidly but to a lower extent, while PA1210WB and TPE10WB samples deformed less and slowly compared with neat polymers.

As expected, the creep recovery behavior was concordant with the creep compliance analysis previously discussed. Rigid and stiff PLA composites recovered 100% of deformation in just 15 min, and the elastic contribution of recovery represented 86% of the final recovery. Soft materials such as TPU-, TPE-, and PA12-based samples are able to recover around 80–90% of deformation, indicating that a certain part of the viscous compliance is permanent. Nevertheless, the addition of WB fibers mitigates this effect since the viscous compliance is lowered. Moreover, because of the creep, deformation is much more marked in WB-based composites, and their unrecovered strain is low in terms of absolute values (and not in percentages). Therefore, it can be stated that the addition of WB fibers, increasing the viscosity of neat polymers, inhibits the slippage of macromolecular chains and, thus, the permanent strain significantly decreases [30].

## 4. Conclusions

After their physical and chemical characterization, WB fibers were melt-mixed at 10 wt.% within PLA, PA12, TPU, and TPE, and the obtained composites were investigated in depth in terms of thermal, thermo-mechanical, morphological, superficial, and viscoelastic properties. Among all formulations, the PLA10WB sample exhibited the best performance, since the tensile modulus, tensile strength, impact resistance, and creep resistance were simultaneously enhanced by the addition of WB fibers. These outstanding results were explained by the excellent chemical and physical affinity between the PLA matrix and WB fibers. Because of its polar nature, PLA exhibited optimal interface adhesion with hydrophilic WB fibers and thus the stress transfer was enhanced. Moreover, this chemical affinity also affected the processing step, in which higher torque values were required to mix the components. As a consequence of this intensive mixing step, WB fibers were well dispersed and distributed within the PLA matrix and they were subjected to de-fibering phenomena. which, in turn, further enhanced the polymer–fiber adhesion and thus the mechanical performance. In other words, the morphological properties observed in PLA10WB were both cause and consequence of the excellent processing step, and these two aspects positively affected the mechanical behavior of the PLA-based composite. In the case of PA12- and TPU-based composites, morphological analysis showed both positive factors, such as partial de-fibering in PA1210WB and good matrix–fiber interfacial adhesion in TPU10WB, and negative factors, such as the presence of voids and a not-optimal distribution and dispersion of WB fibers in the matrices. Therefore, resulting mechanical properties such as tensile strength and elongation at break, which are strongly connected with the morphology of composites, were lower than the ones of neat polymers. Nevertheless, the loss of proprieties was not so dramatic, and further chemical and/or physical treatments of the WB fibers could improve the overall performance. TPE polymer, because of its pronounced hydrophobicity, was not been able to interact sufficiently with WB fibers, and the mechanical behavior of TPE10WB dramatically worsened, especially in terms of elongation at break.

Dynamic mechanical analysis showed that WB fibers can be used as stiffening agents within a wide range of temperatures without significantly affecting the polymer chain mobility, as testified by the damping curves. Once again, only the TPE10WB sample showed an altered behavior: the glass transition of the hard segment of the elastomeric component of TPE, in fact, increased, and this fact could have contributed to the excessive brittleness of the material. In parallel, the investigation of creep compliance and creep recovery behaviors pointed out the ability of WB fibers to enhance both the creep resistance and the deformation recovery of each tested polymer, and this positive behavior could be exploited for applications in which limited creep deformations or complete creep recoveries are needed.

In conclusion, the use of WB wastes as reinforcing fibers is overly satisfactory, and further studies should investigate the effect of higher loading of WB fibers in other polymer matrices, especially hydrophilic ones, in order to obtain new performant composite materials able to mitigate carbon consumption and solve the waste management problems of leather companies.

## Figures and Tables

**Table 1:** Physical parameters and thermal stability data of WB fibers.

Physical Parameters	
Moisture content (wt.%)	20 ± 2
Apparent density (g dm^−3^)	190 ± 3
Real density (g dm^−3^)	300 ± 4
Diameter size (fiber body) (μm)	70 ± 15
Diameter size (coarse clusters) (μm)	222 ± 18
Diameter size (tiny ramifications) (μm)	22 ± 3
**Thermal Stability Data (TGA)**	
T_5_ (°C)	298.8 ± 0.2
T_15_ (°C)	325.5 ± 0.3
T_peak_ (°C)	328.6 ± 0.2
R_600_ (wt.%)	38.4 ± 0.7
R_200;10’_ (wt.%)	98.2 ± 0.4

T_5_ and T_10_ are the temperatures at which 5% and 10% of mass weight decreases are observed; T_peak_ is the temperature at which the mass loss rate is maximum; R_600_ is the mass weight residue evaluated at 600 °C; and R_200;10’_ is the residue evaluated after 10 min of exposition at 200 °C.

**Table 2:** Thermogravimetric analysis (TGA) of the WB-based composites.

	T_5_ (°C)	T_15_ (°C)	T_peak_ (°C)
PLA	334.1	347.4	370.4
PLA10WB	329.2	344.0	363.5
PA12	327.2	329.7	469.0
PA1210WB	287.5	331.2	330.6
TPU	321.1	334.9	415.7
TPU10WB	320.3	334.9	345.4
TPE	370.8	404.0	444.7
TPE10WB	324.5	325.6	426.2

**Table 3:** Thermal properties of WB-based composites evaluated by DSC.

	Cooling Cycle		2nd Heating Cycle						
Sample	T_C_ (°C)	H_C_ (J g^−1^)	T_g_ (°C)	T_pc_ (°C)	H_pc_ (J g^−1^)	T_m_ (°C)	H_m_ (J g^−1^)	χ (%)	Δχ (%)
PLA	-	-	57.8	124.5	15.5	152.5	16.1	0.6	-
PLA10WB	-	-	57.8	127.4	13.2	152.5	13.6	0.5	-
PA12	136.2	32.1	-	-	-	171.9	25.1	12.0	-
PA1210WB	141.1	29.9	-	-	-	170.7	24.1	12.8	+1.5
TPU	106.1	13.3	-	-	-	175.5	10.6	n.a. ^1^	-
TPU10WB	106.8	14.9	-	-	-	173.7	9.1	n.a.	−4.3
TPE	95.1	24.5	-	-	-	145.0	17.4	n.a.	-
TPE10WB	98.7	22.9	-	-	-	144.0	15.8	n.a.	+1.0

^1^ n.a.: not applicable.

**Table 4:** Tensile properties (E, TS, and $\varepsilon_{b}$), Charpy impact properties, and water contact angle values (Θ) of WB-based composites. V_f_ refers to the fiber volume fraction.

Sample	V_f_ (-)	E (MPa)	TS (MPa)	$\varepsilon_{b}$ (%)	Impact Strength (kJ m^−2^)	Θ (°)
PLA	-	1529 ± 108	64.4 ± 0.8	8.7 ± 0.5	14.4	90.6 ± 2.6
PLA10WB	0.31	1803 ± 134	65.3 ± 1.4	5.4 ± 0.3	16.0	110.2 ± 3.5
PA12	-	161 ± 5	34.2 ± 2.1	596 ± 71	nb ^1^	98.9 ± 0.7
PA1210WB	0.27	219 ± 4	22.1 ± 0.3	203 ± 14	nb	105.8 ± 4.3
TPU	-	176 ± 23	42.2 ± 2.0	748 ± 37	nb	119.9 ± 1.3
TPU10WB	0.30	279 ± 16	23.4 ± 1.0	317 ± 49	nb	122.2 ± 2.0
TPE	-	331 ± 4	19.6 ± 1.0	1365 ± 44	nb	124.0 ± 3.4
TPE10WB	0.25	397 ± 26	14.1 ± 1.3	65 ± 1	nb	115.6 ± 3.4

^1^ not broken.

**Table 5:** Qualitative resuming of the morphological properties of WB-based composites.

Sample	Distribution/Dispersion	De-Fibering	Voids	Adhesion
PLA10WB	Excellent	Significant	No	Excellent
PA1210WB	Moderate	Moderate	Few	Moderate
TPU10WB	Scarce	Poor	No	Moderate
TPE10WB	Scarce	Absent	Many	Scarce

**Table 6:** Dynamic mechanical analysis (DMA) of WB-based samples.

	T_g_^1^	E’ (−30 °C)	E’ (0 °C)	E’ (30 °C)	E’ (60 °C)	E’ (90 °C)
	(°C)	(MPa)				
PLA	72.0	3088	2989	2832	2632	14.1
PLA10WB	71.3	2975	2878	2736	2559	23.3
PA12	25.0	1010	660	284	134	70.1
PA1210WB	23.7	1171	793	359	190	113
TPU	41.4	1779	1200	475	140	37.1
TPU10WB	32.8	1889	1254	557	224	70.2
TPE	−7.1/40.4 ^2^	687	195	50.3	90.6	92.3
TPE10WB	−8.4/62.3 ^2^	879	293	127	52.1	68.1

^1^ The glass transition temperature was evaluated as the temperature at which the maximum value of the damping factor (tan δ) was observed. ^2^ TPE-based samples exhibited two glass transition temperatures; the first one refers to the transition of the amorphous domains of the polyolefin and the second one to the relaxation of the hard styrene–olefin block copolymer.

**Table 7:** Creep compliance and recovery strain: extrapolated and empirical data.

	Creep Compliance								
	$J_{10^{\prime }}$	$J_{E}$	$J_{V}$	$J_{\infty }$	t_c_	b_c_	$J_{E}/J_{10^{\prime }}$	$J_{E}/J_{\infty }$	$J_{10^{\prime }}/J_{\infty }$
Sample	(µm²/N)				(s)	(-)	(%)		
PLA	422	365	171	536	3816	0.47	86	68	79
PLA 10WB	400	343	160	503	3125	0.49	86	68	80
PA12	9757	7206	4710	11,913	1030	0.43	74	60	82
PA12 10WB	5480	4289	2294	6583	1221	0.41	78	65	83
TPU	6846	3883	4290	8173	453	0.52	57	48	84
TPU 10WB	4419	2943	1624	4567	163	0.60	67	64	97
TPE	33,781	18,764	16,233	34,997	156	0.63	56	54	97
TPE 10WB	18,749	10,623	9197	19,820	206	0.63	57	54	95
	**Creep Recovery**								
	$R_{15^{\prime }}$	$R_{E}$	$R_{V}$	$R_{\infty }$	**t_c_**	**b_c_**	$R_{E}/R_{15^{\prime }}$	$R_{E}/R_{\infty }$	$R_{15^{\prime }}/R_{\infty }$
**Sample**	**(%)**				**(s)**	**(-)**	**(%)**		
PLA	100	86.4	13.6	100	218	0.72	86	86	100
PLA 10WB	98.6	85.8	14.2	100	328	0.63	87	86	99
PA12	90.5	72.7	18.6	91.3	111	0.53	80	80	99
PA12 10WB	94.0	77.6	17.4	95.0	130	0.52	83	82	99
TPU	91.0	58.4	32.8	91.2	108	0.60	64	64	100
TPU 10WB	87.5	64.3	23.5	87.8	99	0.59	73	73	100
TPE	76.6	46.1	31.4	77.4	124	0.60	60	60	99
TPE 10WB	85.0	54.4	32.6	87.0	146	0.57	64	63	98

## Data Availability

The authors confirm that the data supporting the findings of this study are available within the article.
